# The Differences in Ultrasound and Clinicopathological Features between Basal-Like and Normal-Like Subtypes of Triple Negative Breast Cancer

**DOI:** 10.1371/journal.pone.0114820

**Published:** 2015-03-03

**Authors:** Ziyao Li, Min Ren, Jiawei Tian, Shuangquan Jiang, Yujie Liu, Lei Zhang, Zhenzhen Wang, Qianqian Song, Chong Liu, Tong Wu

**Affiliations:** 1 Department of Ultrasound, Second Affiliated Hospital of Harbin Medical University, Harbin, Heilongjiang Province, China; 2 Department of Biostatistics, College of Public Health of Harbin Medical University, Harbin, Heilongjiang Province, China; University of North Carolina School of Medicine, UNITED STATES

## Abstract

**Purpose:**

The aim of this study was to identify the ultrasound features and clinicopathological characteristics of basal-like subtype of triple negative breast cancers (TNBCs).

**Materials and Methods:**

This study was approved by the ethical board of the Second Affiliated Hospital of Harbin Medical University. The patients’ clinicopathological information was available. The ultrasound features of 62 tumors from 62 TNBC patients were interpreted. The immunohistochemical results of cytokertain5/6 (CK5/6) and Epidermal Growth Factor Receptor (EGFR) were used to classify the tumor into basal-like and normal-like groups. The association of the ultrasound features interpreted by experienced ultrasound doctors with the immunohistochemical classification was studied.

**Results:**

Of the 62 TNBC cases, 42 (67.7%) exhibited the basal-like phenotype and 20 (32.3%) exhibited the normal-like phenotype based on the immunohistochemical CK5/6 and EGFR markers. Of all the tumors, 90.3% were invasive carcinomas. The basal-like tumors were significantly associated with a maximum diameter on ultrasound of more than 20 mm (36, 85.7%) (P = 0.0014). The normal-like tumors usually exhibited lateral shadows (15, 75%) (P = 0.0115) as well as microlobulated margins (12, 60%) (P = 0.0204) compared to the basal-like subtype. Other ultrasound features showed no significant differences between the two groups.

**Conclusions:**

Although ultrasound cannot yet be used to differentiate between the basal-like subtype and normal-like subtype of TNBC, ultrasound can be used to provide some useful information to the clinicians.

## Introduction

Basal-like tumors comprise an important heterogeneous group of breast cancers associated with aggressive behavior and poor prognosis. They account for 15% of all breast cancers and often present as interval cancers that mainly affect younger patients [1–4]. Basal-like breast cancer usually lacks the expression of estrogen receptor (ER), progesterone receptor (PR) and Human Epidermal Growth Factor Receptor 2 (HER-2), which is generally defined as triple negative breast cancer (TNBC). It has been reported that basal-like breast cancers account for 56–85% of TNBCs, and the remainder of TNBCs, which lack the gene expression profile of the basal-like tumors and are similar to normal mammary stromal cells, are normal-like breast cancers [1,2]. Compared to the basal-like subtype, normal-like breast cancer has a very similar or better prognosis to that of hormone receptor positive breast cancers[1,4,5]. Therefore, the sub-classification of basal-like and normal-like TNBCs is essential in determining treatment and estimating the prognosis.

Gene expression profiling (GEP) analysis is the gold standard for diagnosing basal-like breast cancers. However, GEP is still inaccessible in most global clinical settings because of its high cost and high technological and clinical expertise requirements. With this clinical situation, some researchers have proposed a panel of immunohistochemical markers including ER, PR, HER-2, cytokertain5/6 (CK5/6) and Epidermal Growth Factor Receptor (EGFR) to define the basal-like subtype [6–8]; this panel is cost-effective and widely accessible. With the development of high frequency ultrasound, breast ultrasound has been demonstrated to be a useful tool for the differential diagnosis of malignant and benign breast lesions, for guiding breast biopsy and for the guiding therapeutic treatment and prognosis estimation [9]. However, few studies have been conducted to evaluate its clinical value in the differentiation of basal-like and normal-like TNBC subtypes.

In this study, we aimed to identify the clinicopathological and ultrasound features that are correlated with basal-like tumors in 62 TNBC patients with immunohistochemical examination results as proof of the diagnosis.

## Patients and Methods

### Patients

The ethical board of the Second Affiliated Hospital of Harbin Medical University approved our entire study design, methods and verbal consent procedure used in this study. We did not obtain written informed consent because the research was retrospective, non-invasive and all the information about the patients was anonymized. We obtained verbal consent from all of the patients or their next of kin for the use of their data in this study by calling them prior to our study and the ethical board waived the need to document the verbal consent. The breast ultrasound images taken from our prospectively maintained breast tumor database on 62 consecutive patients with TNBCs, who underwent surgery at the Second Affiliated Hospital of Harbin Medical University from June 2012 to December 2013, were used in this study. The inclusion criteria were as follows: 1) single and unilateral breast lesions; 2) the ultrasound examination conducted by the particular sonographer with 3 years of experience, as well as the particular ultrasound machine; 3) standard B-mode and Doppler ultrasound examination available; and 4) immunohistochemical markers (ER, PR, HER-2, CK5/6 and EGFR) accessible. The characteristics of the study subjects are illustrated in Table 1.

**Table 1:** Characteristics of the study subjects.

Variables	Number of patients	Count (%)
Age (years)		
≤ge	45	72.6
>50	17	27.4
Pathological type		
Invasive cancer	56	90.3
*DCIS	6	9.70
Tumor grade for invasive cancer		
Grade 1	3	5.40
Grade 2	22	39.3
Grade 3	31	55.3
Immunohistochemical markers		
EGFR positive	10	23.8
CK5/6 positive	8	19.0
EGFR positive and CK5/6 positive	24	57.2
Lymphatic node metastasis		
Yes	26	41.9
No	36	58.1

*DCIS = ductal carcinoma in situ.

### Breast ultrasound examination

Breast ultrasound examinations were performed by the particular sonographer with 3 years of experience and were interpreted by two sonographers, each with 5 years of experience in the diagnosis of breast cancer. A HITACHI Vision 900 system (Hitachi Medical System, Tokyo, Japan) with high frequency ultrasound and color Doppler features equipped with a 12–5 MHz linear-array transducer was used to perform the breast ultrasound examination. During the real-time examination, longitudinal and transverse static images were obtained from more than two different planes per lesion. Additionally, cine clips in standardized views were also obtained through the masses. In the color Doppler examination, we obtained images and cine clips displaying the most abundant flow signals. Every patient’s static images and cine clips were stored as a separate folder in our work station and then added to our prospectively maintained breast tumor database for subsequent application.

### Histological examination and Immunohistochemistry

The histological examination was conducted by the pathology department at our hospital. All patients were examined to determine the histological tumor type, tumor grade for invasive cancer and lymph node metastasis after the surgery. Invasive cancer was graded as grade 1 (well differentiated), grade 2 (moderately differentiated) or grade 3 (poorly differentiated) according to the Scarff-Bloom-Richardson System [10]. Specimens from the surgically resected lesions were fixed with formalin and embedded in paraffin blocks. Three slices from each patient were selected for immunohistochemistry analyses of membrane and cytoplasm. The immunohistochemical classification was performed by two pathologists each with more than 3 years of experience. In cases of inconsistencies, consensus of the two pathologists was used as the final result.

The appropriate antibodies were chosen for ER, PR, HER2, EGFR and CK5/6 immunohistochemical staining. The cutoff point for ER and PR negative was less than 10% of the cells with nuclear staining [11,12]. HER2 status was graded as 0, 1+, 2+ or 3+, and 0 and 1 + were deemed to be negative HER2 status [13]. Membrane staining was assessed for EGFR according to DAKO criteria. Any intensity of EGFR in more than 1% of cells was considered to be a positive basal marker, and the detection of CK5/6 cytoplasmic expression in either tumor or surrounding tissues was considered to be CK5/6 positive [1]. EGFR positive, CK5/6 positive or EGFR and CK5/6 positive TNBCs were classified as basal-like cancers, and TNBCs that were not positive for those markers were classified as normal-like cancers [1,4,5].

### Interpretation of ultrasound findings

The interpretation of the ultrasound images was performed by two radiologists (each with more than 5 years of experience in the diagnosis of breast cancer) who were blinded to the immunohistochemistry results. In cases of inconsistencies, consensus between the two radiologists was used as the final result.

Features of tumor size (maximum diameter measured by ultrasound), shape, growth orientation, boundary, margin, posterior acoustic echo, lateral acoustic shadow, microcalcifications, and echogenicity were evaluated from the B-mode ultrasound images and categorized using the criteria as illustrated in Table 2. From the color Doppler examination, the blood flow properties of each lesion were scored according to the Alder grading method [14].

**Table 2:** Sonographic features with explanations.

Features	explanations
Tumor size in ultrasound: < 20 mm / ≥20 mm	The size refers to the largest diameter measured on ultrasound.
Tumor shape: Round/Oval/Irregular	The tumor with oval shape may include 2 or 3 undulations, i.e., “gently lobulated.”
Growth orientation: Height >width/ Height ≤ width	It has been reported that tumors with the feature of taller rather than wider are likely to be malignant [10]. In our study, we considered it positive if any part of the lesion was taller than wide.
Boundaries: abrupt interface/echogenic halo	An abrupt interface indicates that there is no echogenic transition zone that is considered a sign of invasion between the lesion and the surrounding tissue[16].
Margins: Circumscribed/Not circumscribed: indistinct, microlobulated, angular, speculated	The mass with a non-circumscribed margin may be indistinct, microlobulated, angular, or speculated.
Posterior shadowing: Yes/no	Posterior shadowing is associated with fibrous tissue in the tumor and is often observed in low-grade infiltrating ductal carcinomas and tubular carcinomas, maybe as they grow slowly enough to allow the intensely shadowing desmoplastic reaction to occur[9,16].
Lateral acoustic shadow: Yes/no	The lateral acoustic shadow is always produced by the capsule on both the inner and outer surface of the tumor. The capsule is commonly considered to be the tumor pushing rather than being invasive to the surrounding tissue [16,28,30].
Microcalcification: Yes/no	The microcalcification refers to the diameter of less than 0.5 mm in the mass in this study.
Echogenicity: Hypo-echo/mixed echo	We defined the echogenicity of the tumor compared with the fat in the breast[16]. The mixed echo in our study was defined as three conditions as follows: marked hypoechoicity surrounding the central hyperechoicity or isoechoicity, all types of echoicities intertwining, the central marked hypoechoicity with peripheral lesion hyperechoicity or isoechoicity.
Density of the flow signals: 0–4	The flow signals were evaluated according to the Adler blood grade, which divided the blood flow into 4 levels; levels 0 and 1 indicate unrich, whereas levels 2 and 3 indicate abundant[14].

### Statistical analyses

Chi-squared tests or Fisher exact tests were performed on each single clinicopathological or qualitative ultrasound feature of the two TNBC subtypes to evaluate the significance of difference. For correlations, we performed multivariate regression analysis and expressed the odds ratio (OR) with 95% confidence intervals (CI). The P value was two sided, and a P value ≤0.05 indicated a significant difference.

SPSS software (version 21.0; Chicago, IL, USA) was used for the statistical analysis.

## Results

### Clinicopathological and immunohistochemistry

In total, 62 patients were analyzed in this study including 48 invasive ductal carcinomas, 6 ductal carcinomas in situ, 3 medullary carcinomas, 3 metaplastic carcinomas, 1 clear cell carcinoma, and 1 apocrine carcinoma. All patients were female and the ages ranged from 32 to 71 years (mean age, 48.8 years). The age range of the 42 patients with basal-like subtype was 32–71 years (mean age, 49.4 years), whereas the age range of the 20 patients with normal-like subtype was 38–63 years (mean age, 47.6 years). No significant age differences were observed between the two sub-groups (P = 0.223) or based on the histological tumor type (P = 0.377), the tumor grade of invasive cancer (P = 0.410) and lymphatic metastasis (P = 0.445). The detailed information is shown in Table 3.

**Table 3:** The patients’ clinical pathological characteristics in the two groups.

	Age (Y)		The histological type		The tumor grade in invasive cancer		Lymphatic metastasis	
	≤ym	>50	Invasive carcinoma	DCIS	Grade 1–2	Grade 3	Yes	No
Basal-like	28 (67%)	14(33%)	39(93%)	3(7%)	16(41%)	23(59%)	19 (45%)	23 (55%)
Normal-like	17 (85%)	3(15%)	17(85%)	3(15%)	9(53%)	8(47%)	7 (35%)	13 (65%)
P value	0.223		0.377		0.410		0.445	

Among the 62 TNBCs, 42 were classified as basal-like tumors and 20 were classified as normal-like tumors. In the 42 basal-like tumors, 23.8% (10/42) were EGFR positive only, 19.0% (8/42) were CK5/6 positive only and 57.2% (24/42) were both EGFR and CK5/6 positive.

### Ultrasound findings

The results of the chi-squared test and multivariate regression analysis for the ultrasound findings are shown in Tables 4 and 5. Forward selection was used in the multivariate regression models comparing the ultrasound findings of the two subtypes to screen the variables, and the standard was P value ≤0.05. The selected parameters include tumor margin, posterior feature, lateral shadow and tumor size. The result of multivariate regression analysis is shown in Table 5.

**Table 4:** The result of chi-squared test of the ultrasound features by groups.

Group	Basal-like	Normal-like	P
Tumor size			
<20mm	6	13	0.000**
≥20mm	36	7	
Growth orientation			
Aspect ratio>1	8	10	0.012**
Aspect ratio<1	34	10	
Lateral acoustic shadow			
Yes	19	15	0.028**
No	23	5	
Tumor shape			
Round/oval	11	8	0.270
Irregular	31	12	
Echo pattern			
Hypoechoic	9	4	1.000
Mixed/marked hypoechoic	33	16	
Tumor margin			
Microlobulated	15	12	0.071
Angular/speculated	27	8	
Posterior acoustic feature			
No change	20	12	0.057
Enhancement	18	3	
Attenuation	4	5	
Microcalcification			
Yes	29	11	0.280
No	13	9	
Boundary			
Echogenic halo	0	2	0.100
Abrupt interface	42	18	
Blood flow signals			
Alder 0–1	16	4	0.154
Alder 2–3	26	16	

Note: ** indicates significant difference in the two groups.

**Table 5:** The result of the multivariate logistical regression analysis.

variables	SE	χ^2^	P-value	OR	OR (95%CI)
margin	0.9707	5.3757	0.0204	9.494	(1.416,63.641)
Posterior feature	1.5397	3.3286	0.0681	0.060	(0.003,1.232)
Lateral shadow	1.0045	6.3904	0.0115	12.670	(1.769,90.732)
size	0.9857	10.1599	0.0014	23.147	(3.353,159.785)

**Fig 1:**
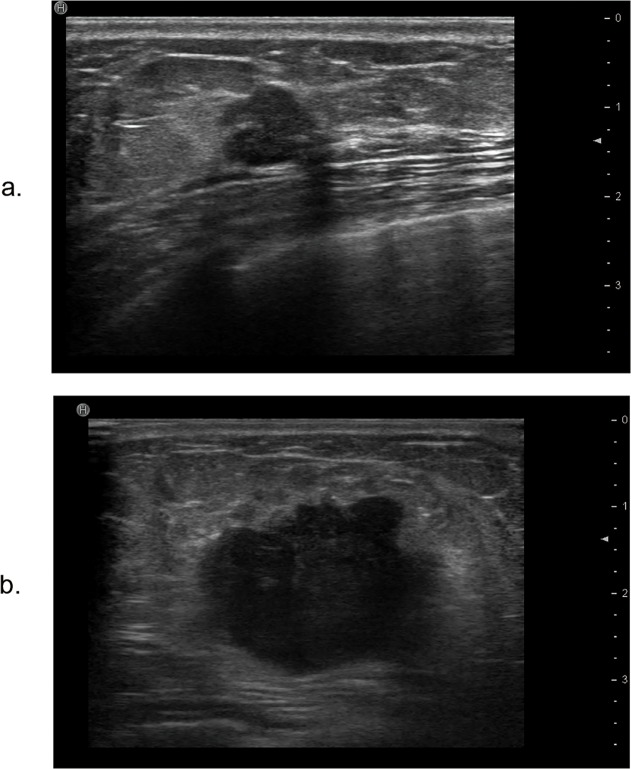
The maximum diameter of the tumor and lateral acoustic shadow feature on ultrasound. a. The maximum diameter of the normal-like breast cancer is 14 mm, and the lateral acoustic shadow is shown. b. The maximum diameter of the basal-like breast cancer is 30 mm. There is no lateral acoustic shadow.

**Fig 2:**
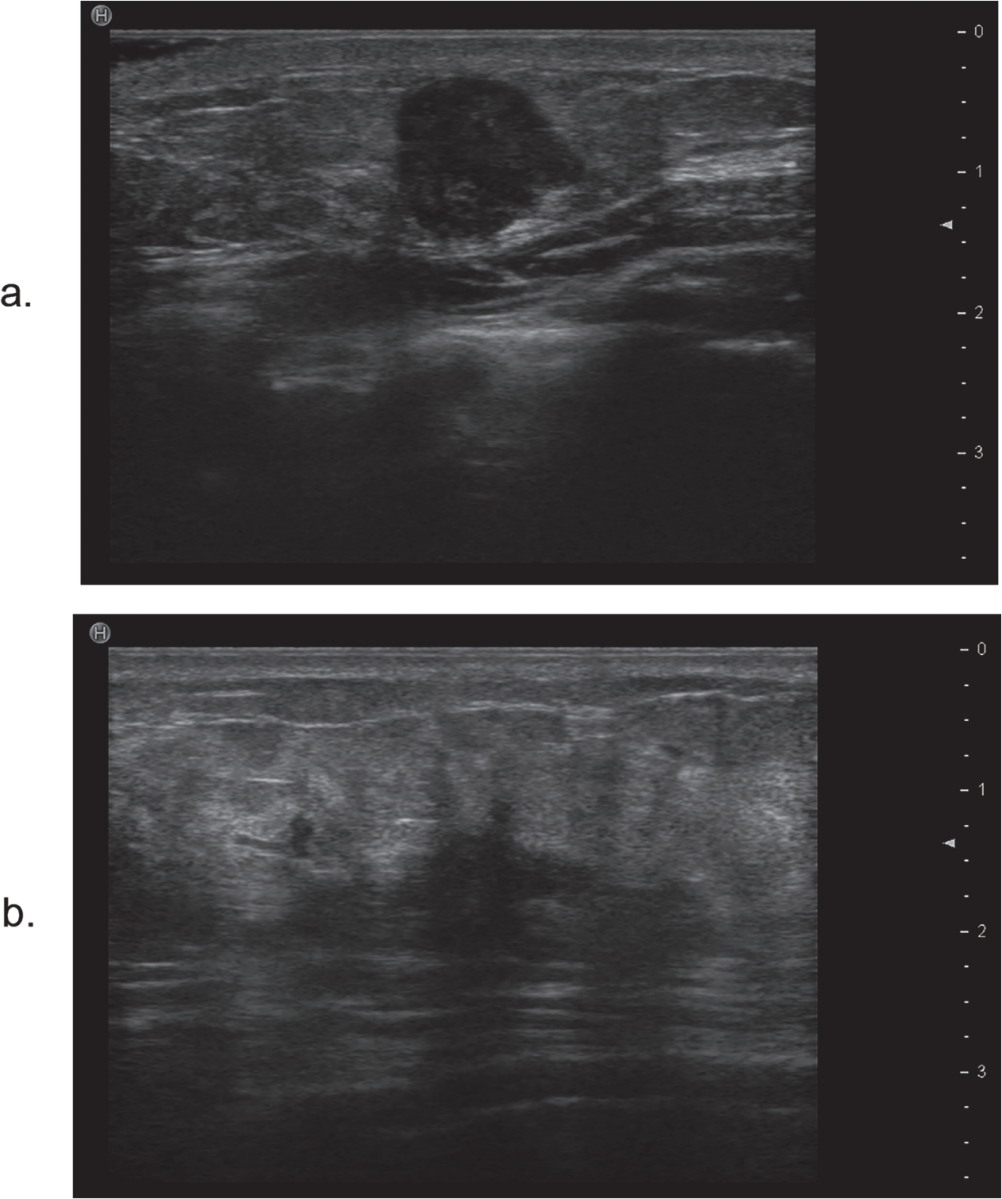
The tumor margin on ultrasound. a. It shows that there is a slight lobulated margin of normal-like breast tumor. b. The ultrasound image of a basal-like breast cancer shows that the tumor margin is angular and speculated.

**Fig 3:**
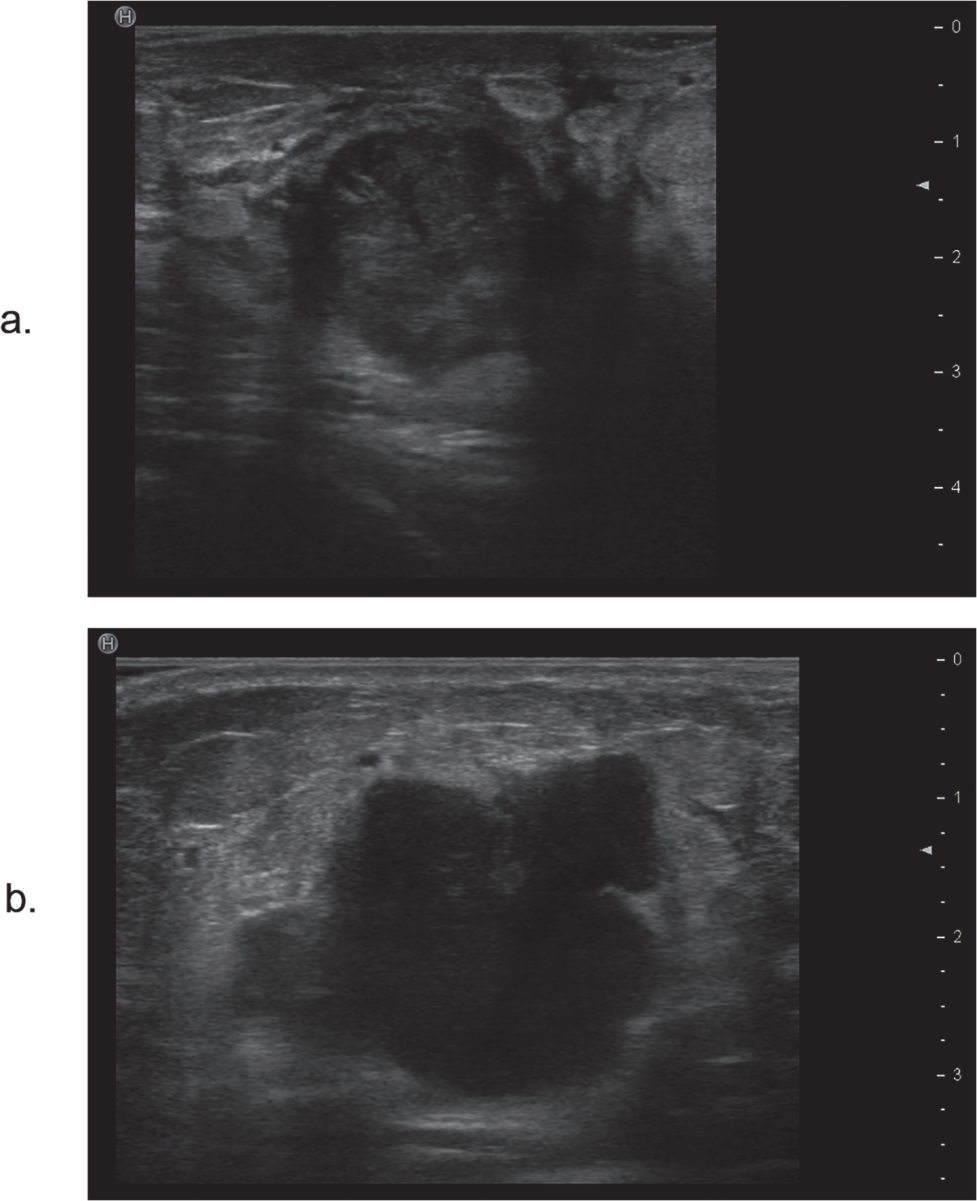
The relationship between lateral acoustic shadow feature on ultrasound and molecular subtypes of breast cancer. a. The ultrasound image of a tumor with lateral acoustic shadow is classified as the normal-like subtype according to the immunohistochemistry results. b. The ultrasound image of a tumor without lateral acoustic shadow is classified as the basal-like subtype according to the immunohistochemistry results.

Regarding the ultrasound findings of the two groups, significant differences were observed in the tumor maximum diameter measured on the ultrasound, the tumor margin and lateral acoustic shadow. Tumor size ≥20 mm was significantly more frequent in the basal-like group than in the normal-like group (85.7% vs 35%) (P = 0.0014) (see Fig. 1). According to the results of the analyzed images, we noticed that there were no tumors with circumscribed or indistinct margins in our study. An angular/speculated margin was more common in basal-like tumors (64.3% of the basal-like subtype versus 40% of the normal-like subtype) (P = 0.0204) (see Fig. 2). There were 19 basal-like subtype cases with lateral shadow and 15 normal-like subtype cases with lateral shadow (45.2% vs 75%) (P = 0.0115) (see Fig. 1 and Fig. 3).

The growth orientation of the tumor in the two subtypes had significant difference based on the chi-squared test (P = 0.012), but it was not involved in the multivariate regression model when we screened variations. So there was no significant difference in multivariate regression analysis for the tumors’ growth orientation.

**Fig 4:**
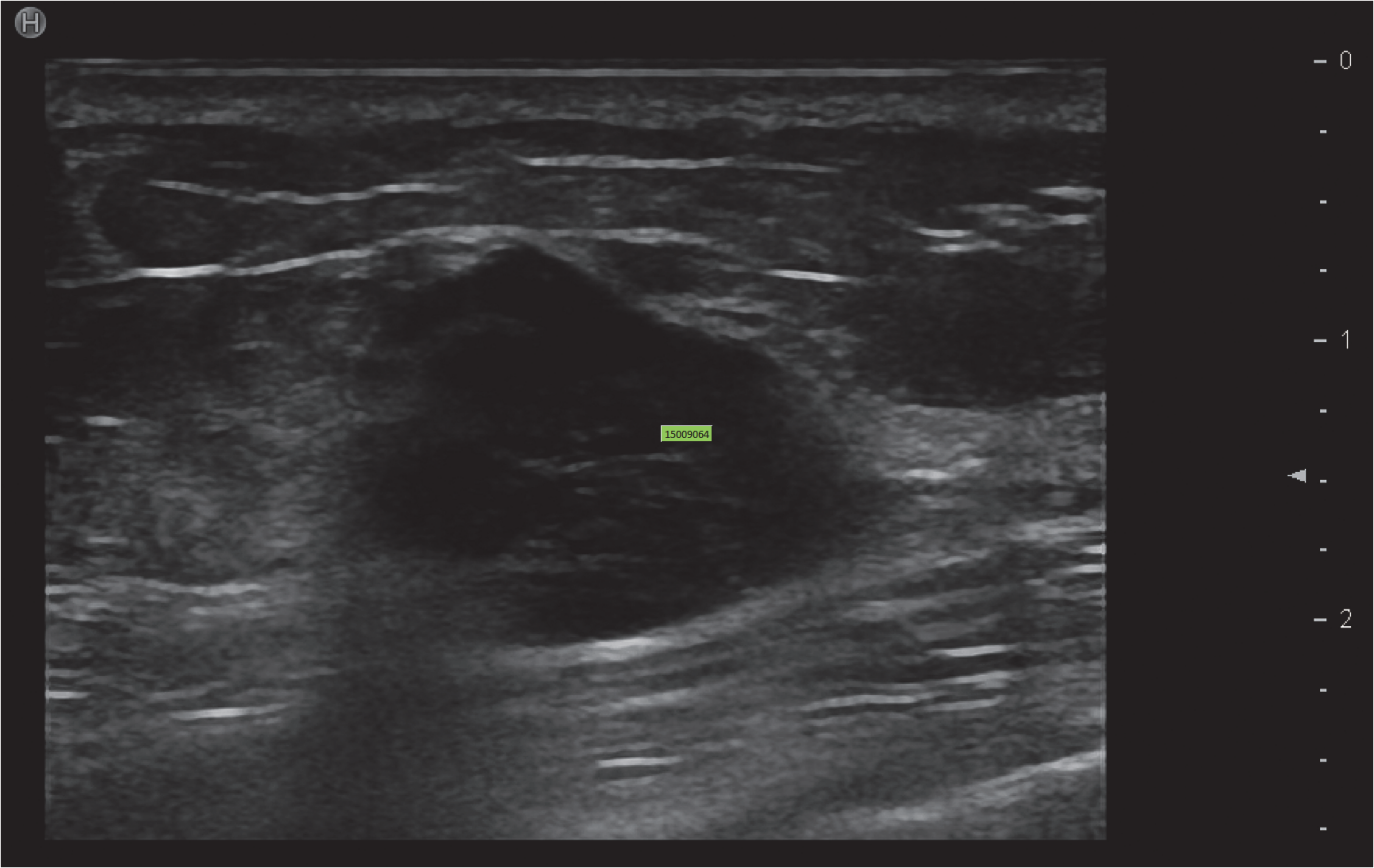
The echo pattern of the breast tumor with TNBC subtype. We observed the marked hypoechoicity in the tumor, and all three markers including ER, PR, and HER2 are negative.

It has been reported that the TNBCs had a tendency to show marked hypoechoicity [15]. In our study, based on the image analysis, we defined the lesion mixed echo as the three conditions explained in Table 2. The echo pattern of the tumors in our research all showed hypoechoicity or mixed echoicity, and none showed hyperechoicity or isoechoicity. A total of 49 (79%) tumors showed mixed echoicity, 33 (78%) were basal-like cancer and 16 (80%) were normal-like cancer. Based on the echo pattern, the TNBC always presents as predominant hypoecho with other mixed echo patterns (see Fig. 4). Other ultrasound features, including the tumor shape, posterior acoustic feature, microcalcifications, boundary, and blood flow signal, had no significant differences between the two groups.

## Discussion

Ultrasound and mammography are two vital imaging tools for breast screening. Ultrasound is particularly important as the mammography has some limitations. It can be used to distinguish the malignant from benign lesions, to guide biopsies, to assist in the selection of the appropriate therapeutic method and to aid further management [9,16,17]. Currently, TNBC has become a top global issue. A series of studies have shown that TNBC is a subtype that has a more aggressive clinical course compared with other forms of breast cancer and that is associated with relatively high rates of recurrence, distant metastases and low overall survival rates [15,18–20]. The TNBC and basal-like subtype are usually thought to be identical based on clinical work-up, but this is not actually true [21,22]. Gene expression profiling (GEP) analysis is the gold standard for diagnosing basal-like breast cancer, but currently, the use of GEP is still limited due to financial concerns and because this technology is often inaccessible in most global clinical settings. Given these circumstances, some researchers have proposed a panel of immunohistochemical markers defining the basal-like subtype, including ER, PR, HER-2, CK5/6 and EGFR, with a sensitivity of 55–76% and a specificity of 100% [6–8]. With this method, the TNBCs are divided into the basal-like subtype and the normal-like subtype. Previous studies have proved that basal-like breast cancers are particularly more aggressive than the normal-like tumors [1,4,5,7]. We sought to demonstrate whether there is a difference in the ultrasound and clinicopathological features between the basal-like subtype and the normal-like subtype of TNBC. Although we cannot differentiate the molecular subtypes of breast cancers based on ultrasound imaging, the radiologists should be aware of different subtypes with distinctive ultrasound features that provide more information to clinicians.

Many studies reported that patients with TNBC were younger than those without TNBC [5,18,19,23–25]. In all 62 patients enrolled in our study, the number of patient ≤50 years old accounted for 72.6%, and so there was a clear predominance of younger patients. However, this discrepancy in the age of onset did not exist in the basal-like and normal-like cancers in our study.

As one of the main prognostic indicators as well as a determining factor for surgical treatment planning, the breast cancer size is vital. It is reported that the tumor size measured on ultrasound has a comparatively good correlation with the tumor size on pathology [26]. Therefore, accurate prediction of tumor size based on ultrasound is essential. Regarding the tumor’s maximum diameter measured by ultrasound, the basal-like tumors were usually larger than the normal-like subtype. This may be relative to the highly malignant and invasive nature of basal-like breast cancers. Due to these biological behaviors, the tumors in this subtype always grow fast and may rapidly lead to an invasive stage and present with a larger volume.

Other ultrasound features that were found to be significantly different in the two groups in our study were the tumor margin and lateral acoustic shadow. The angular/speculated margin and no lateral acoustic shadow were more common in the basal-like breast cancers. An angular/speculated margin always indicates that the tumor grows fast and that the growth speed in every direction is not consistent. The lateral acoustic shadow is thought to originate from the capsule, which is on both the tumor’s inner and outer surfaces and is usually a pseudocapsule of compressed adjacent normal breast tissue. Visualization of such a capsule always indicates that the leading edge of the nodule is pushing rather than infiltrating [16]. Therefore, the result of our study was consistent with the property of basal-like and normal-like breast tumors that the basal-like subtype is usually more malignant than the normal-like subtype and it always invasive rather than just encroaching.

The growth orientation was found to be different in the two groups based on the chi-squared test result. However, the difference on the multivariate logistical regression was not significant. It has been reported that the malignant nodules are more likely to present with masses that are taller than wider [16,27–29]. This finding is alarming because it suggests growth across normal tissue planes, which are horizontally oriented in patients scanned in the supine position [16,27]. The result of our study demonstrated that an aspect ratio >1 may reduce the possibility of a tumor being a basal-like cancer, but it is not an independent influencing factor. Other ultrasound features had no significant differences between the two groups.

There are certainly some limitations to our research, including the retrospective nature of the study. Another important limitation is that because TNBCs account for only 10–15% of all breast cancers and the basal-like subtype or normal-like subtype accounts for a lower proportion, the limited number of patients in our cohort is unavoidable. Therefore, we are still in the process of acquiring cases to further support these conclusions. In addition, one of the purposes of this study is to attract people’s attention to these two subtypes of breast cancer, so that more people can develop more comprehensive and in-depth exploration in the future. Also, we didn’t test the predictive ability of these distinguishing ultrasound features between the two subtypes and this would be the emphasis of the next step of our study.

## Conclusions

Although ultrasound cannot yet be used to differentiate between basal-like subtype and normal-like subtype of TNBC, we have demonstrated some sonographic features with a significant difference. Further study is needed to confirm our findings and to explain the biological reasons for these findings.
